# A new species of *Campoletis* Förster (Hymenoptera, Ichneumonidae) with a key to species known from China, Japan and South Korea

**DOI:** 10.3897/zookeys.1004.57913

**Published:** 2020-12-16

**Authors:** Ya-Wei Wei, Yong-Bin Zhou, Qing-Chi Zou, Mao-Ling Sheng

**Affiliations:** 1 College of Forestry, Shenyang Agricultural University, 120 Dongling Road, Shenyang 110866, China Shenyang Agricultural University Shenyang China; 2 Research Station of Liaohe-River Plain Forest Ecosystem, Chinese Forest Ecosystem Research Network, Changtu, Liaoning, 112500, China Chinese Forest Ecosystem Research Network Changtu China; 3 Liaoning Natural Forest Protection Center, 126 Changjiang Street, Shenyang 110036, China Liaoning Natural Forest Protection Center Shenyang China; 4 General Station of Forest and Grassland Pest Management, National Forestry and Grassland Administration, 58 Huanghe North Street, Shenyang 110034, China National Forestry and Grassland Administration Shenyang China

**Keywords:** Campopleginae, taxonomy, parasitoid wasp

## Abstract

A new species of the genus *Campoletis* Förster, 1869, *C. deserticola* Sheng & Zhou, **sp. nov.**, collected from Zhangwu, Liaoning Province and Songshan National Natural Reserve, Yanqing, Beijing, China, is described and illustrated. A taxonomic key to the species of *Campoletis* known in China is provided.

## Introduction

*Campoletis* Förster, 1869, a relatively large genus of the subfamily Campopleginae (Hymenoptera, Ichneumonidae), comprises 112 described species (Yu et al. 2016; Riedel 2017; Vas 2019a, b), of which 23 are from the Eastern Palaearctic region (Kokujev 1915; Kusigemati 1972; Horstmann 1979; Yu et al. 2016; Vas 2019a) (14 of them are found across the whole Palaearctic and three of them are found in both the Eastern Palearctic and the Oriental), seven are from the Oriental region (Gupta 1974; Kusigemati 1987, 1990; Yu et al. 2016), 41 from the Western Palaearctic region (Yu et al. 2016; Riedel 2017; Vas 2019b), seven from the Neotropical region, 50 from the Nearctic region (Yu et al. 2016), and three species are from the Australasian region (Cameron 1903, 1911; Crosby 1994; Yu et al. 2016).

The Western Palaearctic species of *Campoletis* were revised by Riedel (2017); subsequently, three new species of *Campoletis* have been described by Vas (2019a, b) from Mongolia and South-Eastern Europe. Prior to this publication seven species of *Campoletis* had been known from China (Kokujev 1915; Uchida 1957; Kusigemati 1990; He et al. 1996; Sheng and Sun 2014).

The Project “Research Station of Liaohe-River Plain Forest Ecosystem, Chinese Forest Ecosystem Research Network”, set in the desert area in Liaoning Province, has being undertaken by Y-WW’s research group since 2014. One of the purposes of the investigation is recording biodiversity. Large numbers of ichneumonids were collected and in the present research, a new species of *Campoletis* is described which was collected in the desert. With paratypes collected from Yanqing, Beijing, it is described and illustrated herein, and compared with its congeners.

## Materials and methods

### Institutional abbreviations

**GSFGPM** General Station of Forest and Grassland Pest Management, National Forestry and Grassland Administration, China;

**HUM**Hokkaido University Museum, Sapporo, Japan;

**RSLPFE** Research Station of Liaohe-River Plain Forest Ecosystem, Chinese Forest Ecosystem Research Network, Changtu, Liaoning, China.

### Specimen collection

**Figure 1.**
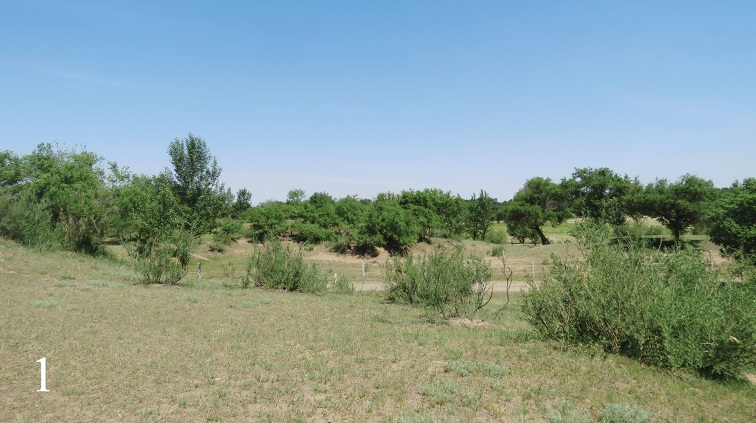
Habitat of *Campoletis deserticola* Sheng & Zhou, sp. nov., the holotype locality in Zhangwu County, Liaoning Province.

Specimens were collected with interception traps (IT) as described by Li et al. (2012) in RSLPFE (Fig. 1) and in the Songshan National Natural Reserve,Yanqing, Beijing.

The forest in RSLPFE are mainly comprised of *Caragana korshinskii* Kom., *Ulmus pumila* L., *Salix matsudana* Koidz., Crataegus pinnatifida var. major N. E. Brown, *Populus* sp., *Lespedeza bicolor* Turcz. The forest floor is covered by *Allium macrostemon* Bunge, *Echinochloa crusgali* (L.) Beauv. and *Medicago sativa* L.

The forest in Songshan National Natural Reserve, Yanqing, Beijing, hold *Ulmus pumila* L., *Salix* spp., *Lespedeza bicolor* Turcz., Vitex negundo var. heterophylla (Franch.) Rehd., *Platycladus orientalis* (L.) Franco, *Pinus tabulaeformis* Carr.

The holotypes of *Campoletis chlorideae* Uchida, 1957 and *Tranosema rugosipropodeum* Uchida, 1942 deposited in the HUM were examined.

Morphological terminology is mostly based on Broad et al. (2018). Images were taken using a Leica M205A stereo microscope with LAS Montage MultiFocus. Type specimens are deposited in the Insect Museum, GSFGPM.

## Taxonomy

### Campoletis

Taxon classificationAnimaliaHymenopteraIchneumonidae

Förster, 1869

B7BCFB12-38F3-57A4-B8DA-AED708C881FD

Campoletis Förster, 1869:157. Type-species: Mesoleptus tibiator Cresson.

#### Diagnosis

**(from Townes 1970; Gupta 1974; Riedel 2017).** Inner margin of eye slightly indented opposite antennal socket. Apical margin of clypeus usually with a median tooth. Lower margin of mandible with narrow lamella, lower tooth of mandible slightly narrower than upper tooth, same length or slightly shorter. Malar space 0.5–1.0× as long as basal width of mandible. Areolet receiving 2m-cu usually basad of middle. Nervellus intercepted; discoidella almost unpigmented, reaching nervellus. Lateral suture between tergite 1 and sternite 1 distinctly below mid-height. Glymma present and deep. Ovipositor 1.6 to 3.5 as long as apical depth of metasoma.

### Key to the *Campoletis* known in China, Japan and South Korea

**Table:** 

1	Fore wing vein 2m-cu vertical or almost vertical, lower-posterior angle of second discal cell right-angled or almost right-angled	**2**
–	Fore wing vein 2m-cu distinctly inclivous, lower-posterior angle of second discal distinctly acute	**3**
2	Antenna stout, second flagellomere approximately as long as wide. Propodeum indistinctly areolated, costula absent. Tegula black	***C. longicalcar* (Kokujev)**
–	Antenna slender, second flagellomere longer than width. Propodeum distinctly areolated, costula present. Tegula yellowish white	***C. tibetana* (Kokujev)**
3	Areolet sessile, receiving vein 2m-cu basad of middle. Head, mesosoma, all tergites and all coxae entirely black. Tegula light yellow	***C. rugosipropodeum* (Uchida)**
–	Areolet with distinct petiole, receiving vein 2m-cu at or basad of middle. Head, mesosoma, tergites and coxae not entirely black. Tegula black, or yellow to brown	**4**
4	Costula absent. Tergite 2 as long as tergite 3. Area superomedia combined with area petiolaris. Tegula yellow	***C. imperfecta* (Kokujev)**
–	Costula present. Tergite 2 longer than tergite 3. Area superomedia separated from area petiolaris by carina, at least junction between them discernible. Tegula black, or yellow to brown	**5**
5	Malar space 1.2× as long as basal width of mandible. Areolet receiving 2m-cu basad of middle. Area basalis reversed triangular	***C. hongkongensis* Kusigemati**
–	Malar space as long as or shorter than basal width of mandible. Areolet receiving 2m-cu at or almost at middle, or area basalis trapezoidal (Fig. 11)	**6**
6	Tergite 2 at most as long as its posterior width	**7**
–	Tergite 2 at least 1.25× as long as its posterior width	**9**
7	Occipital carina complete. Frons without median longitudinal carina; Notaulus almost entirely absent. Ovipositor sheath 1.1–1.2× as long as second tergite. All coxae black. Tegula brown	***C. deserticola* Sheng & Zhou, sp. nov.**
–	Lower portion of occipital carina incomplete. Frons with median longitudinal carina; Notaulus present. Ovipositor sheath 0.8× as long as second tergite. Fore and mid coxae yellow brown, at least not entirely black. Tegula whitish yellow	**8**
8	Lower tooth of mandible shorter than upper tooth. Areolet receiving 2m-cu basad of middle. Area superomedia combined with area petiolaris, without carina between them. subbasal and apical portions of hind tibia blackish, median portion whitish	***C. takizawai* Kusigemati**
–	Lower tooth of mandible as long as upper tooth. Areolet receiving 2m-cu at middle. Area superomedia separated from area petiolaris by distinct carina. Hind tibia entirely yellowish brown	***C. gastrolinae* Kusigemati**
9	Ovipositor sheath 0.64× as long as hind tibia. Hind femur red. Posterior portion of tergite 3 and subsequent tergites reddish brown	***C. chlorideae* Uchida**
–	Ovipositor sheath at most 0.5× as long as hind tibia. Basal and apical portions of hind femur black. Tergites black, at most sides of tergites 3–7 more or less reddish	***C. annulata* (Gravenhorst)**

### Campoletis deserticola

Taxon classificationAnimaliaHymenopteraIchneumonidae

Sheng & Zhou sp. nov.

0FBFD656-B250-562E-8BA7-DB55AB11BC9B

http://zoobank.org/018A514B-3CBB-447B-9A91-E130F1E817AE

Figures 2–14 , 15

#### Material examined.

***Holotype***: China • ♀; Liaoning, Zhangwu, Aershan; 273 m; 18.VI.2020; Ya-Wei Wei leg. ***Paratypes***: China • 2♀♀23♂♂; Beijing, Yanqing, Songshan National Natural Reserve; 672 m; 17–26.IX.2011; IT by Shi-Xiang Zong leg. • 1♀1♂; Liaoning, Zhangwu, Aershan; 273 m; 18.VI.2020; Ya-Wei Wei leg.

**Figures 2–14.**
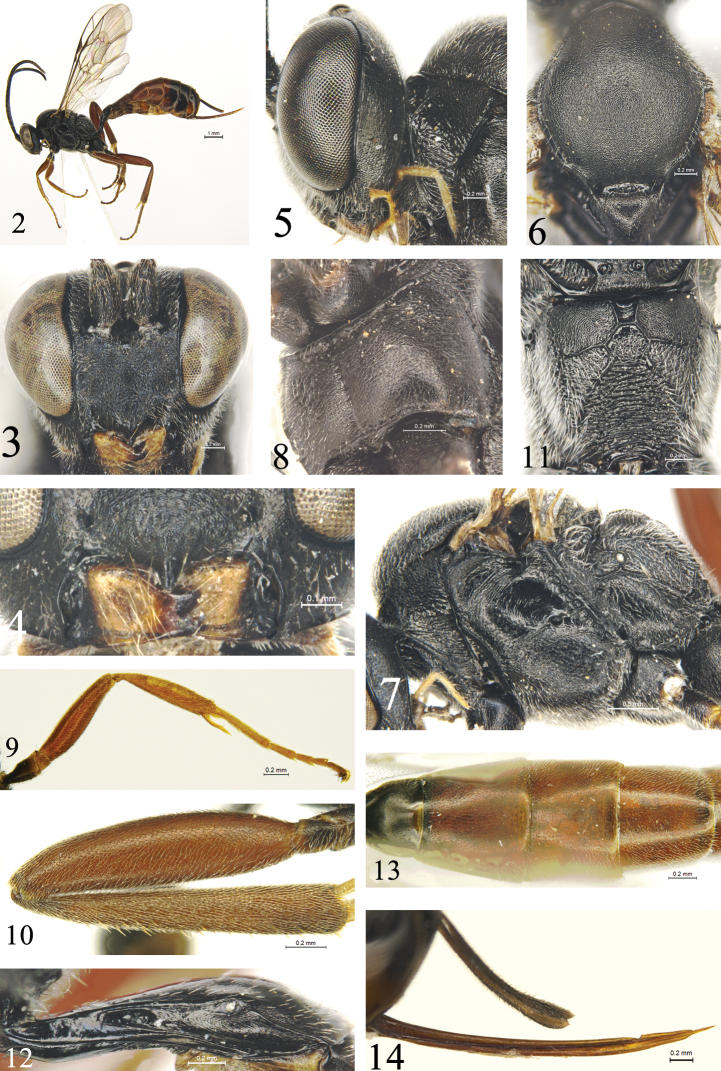
*Campoletis deserticola* Sheng & Zhou, sp. nov. Holotype, female **2** habitus, lateral view **3** head, anterior view **4** clypeus and mandibles **5** head and pronotum, lateral view **6** mesoscutum and scutellum **7** mesosoma, lateral view **8** mesosoma, ventrolateral view **9** fore leg, lateral view **10** hind femur and tibia, lateral view **11** propodeum **12** first tergite, lateral view **13** postpetiole and tergites 2–4, dorsal view **14** ovipositor and ovipositor sheath, lateral view.

#### Diagnosis.

*Campoletis deserticola* Sheng & Zhou, sp. nov. can be distinguished from all other species of *Campoletis* by combination of the following:

Body granulate to finely granulate, without evident punctures. Face (Fig. 3) and main portion of clypeus rough, with indistinct, irregular fine punctures. Malar space 0.8–0.9× as long as basal width of mandible. Mesoscutum and scutellum (Fig. 6) with even leathery culpture. Anterolateral portion of postscutellum with deep circular depressions (Fig. 11). Propodeum (Fig. 11) with dense gray setae; area externa roughly shagreened; area petiolaris with dense transverse wrinkles. Head except mandible, mesosoma and all coxae entirely black. All femora brownish red. Tergites 2–4 almost entirely reddish brown.

#### Description.

**Female.** Body length 6.4–7.2 mm. Fore wing length 4.7–5.0 mm. Ovipositor sheath length 0.9–1.1 mm.

***Head*.** Inner margins of eyes slightly convergent ventrally. Face (Fig. 3) 1.4× as wide as long from dorsal margin to clypeal fovea, evenly slightly convex, rough, with indistinct irregular fine punctures; dorsal margin with a V-shaped concavity medially. Clypeal suture entirely absent. Basal portion of clypeus (Figs 3, 4) with texture as that of face; apical margin smooth, shiny, with strong median tooth. Mandible (Fig. 4) distinctly narrowed to ends of teeth, with sparse brownish yellow setae; upper tooth as long as lower tooth. Malar area, gena (Fig. 5) and vertex shagreened. Malar space 0.8–0.9× as long as basal width of mandible. Gena distinctly convergent posteriorly, in dorsal view 0.5× as long as width of eye. Postocellar line 1.2× as long as ocular-ocellar line. Frons with texture as that of face. Antenna with 28–30 flagellomeres. Ratios of lengths from first to fifth flagellomeres 1.5:1.2:1.1:1.1:1.0. Occipital carina complete, reaching hypostomal carina above base of mandible.

***Mesosoma*.** Lateral concavity of pronotum (Figs 5, 7) wide, shallow, lower half with distinct oblique wrinkles; dorsoposterior portion shagreened. Epomia present. Mesoscutum (Fig. 6) with leathery culpture, evenly convex; notaulus absent. Scutoscutellar groove almost smooth, shiny. Scutellum slightly convex, with fine leathery culpture. Postscutellum shagreened, anterolateral portion with deep circular depressions (Fig. 11). Mesopleuron (Fig. 7) with leathery culpture; in front of speculum slightly concave with longitudinal wrinkles; upper anterior portion with oblique longitudinal wrinkles. Upper end of epicnemial carina reaching anterior margin of mesopleuron, at 0.5 height of posterior margin of pronotum; ventral part (Fig. 8) complete, strongly elevated. Metapleuron slightly convex, with texture as lower portion of mesopleuron; lower-posterior portion with short oblique wrinkles. Ratio of length of hind tarsomeres from first to fifth approximately 5.6:2.4:1.6:1.0:1.3. Claw with two or three teeth. Wings slightly brownish, hyaline. Fore wing with vein 1cu-a distal to M&RS by approximately 0.2× length of 1cu-a; 1cu-a distinctly inclivous. Areolet quadrilateral, with distinct petiole, receiving vein 2m-cu at approximately 0.4 distance from vein 2rs-m to 3rs-m; 2rs-m almost as long as 3rs-m. Postnervulus strongly inclivous, intercepted at lower 0.3. Hind wing vein 1-cu 1.5× as long as cu-a. Propodeum (Fig. 11) with dense gray setae; area basalis reversed trapezoid, shiny, almost smooth; area externa roughly shagreened; area superomedia rough with indistinct fine punctures; area dentipara with indistinct oblique wrinkles; area petiolaris with dense, distinct transverse wrinkles. Area superomedia and area petiolaris confluent, junction between them discernible. Propodeal spiracle small, almost circular, connecting pleural carina by ridge.

***Metasoma*.** Metasomal tergites weakly shagreened. First tergite (Fig. 12) 2.2–2.3 × as long as its apical width, anterior portion and posterior margin smooth, shiny; spiracle small, circular, located at apical 0.33. Second tergite (Fig. 13) trapezoidal, 1.4× as long as its anterior width, 0.9 × as long as its posterior width; longer than third tergite. Third tergite approximately 0.7× as long as posterior width, sides parallel. Ovipositor sheath 0.55–0.65× as long as hind tibia, 0.85–0.95× as long as first tergite, 1.1–1.2× as long as second tergite. Ovipositor (Fig. 14) slightly curved upwards.

***Coloration*** (Fig. 2). Black, except for following: mandible except teeth yellow brown. Apical three segments of maxillary palpi, tegulae and lateral margins of tergite 7 brown. Femora, sides of fore tibia (Fig. 9), hind tibia (Fig. 10) except base, main portion of tergite 2, tergites 3 and 4 and anterior portion of tergite 5 brownish red. Inner side of fore tibia and fore tarsus yellowish brown. Mid tarsus brown. Base of hind tibia and tarsus brownish black. Pterostigma and veins brown.

**Figure 15.**
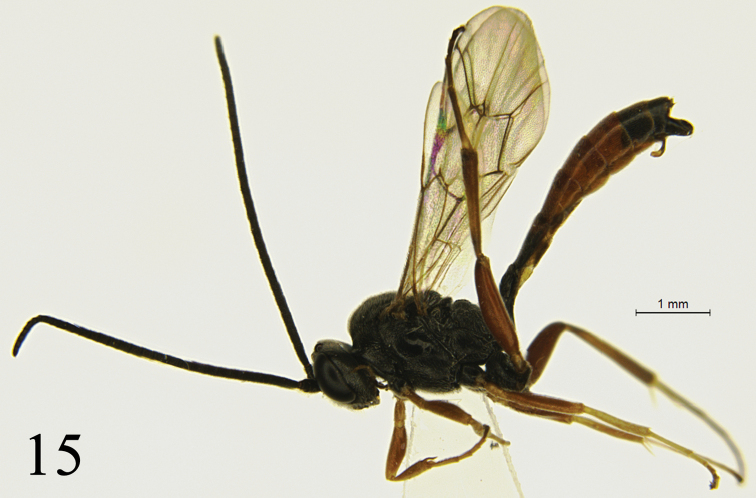
*Campoletis deserticola* Sheng & Zhou, sp. nov. Paratype, male. Habitus, lateral view.

**Male** (Fig. 15). Body length 6.0–7.2 mm. Fore wing length 4.3–5.0 mm. Antenna with 31–34 flagellomeres. Similar to female, except following: fifth tergite almost entirely brownish red; posterior median portion of sixth tergite brown.

#### Distribution.

China: Beijing, Liaoning.

#### Etymology.

The specific name is derived from the habitat of the holotype locality.

#### Remarks.

The new species is similar to *Campoletis gastrolinae* Kusigemati, 1972 and *C. cognata* (Tschek, 1871) in having the head and mesosoma black; tergites 2–3 red to reddish brown; hind femur completely or predominantly red; apical margin of clypeus with strong median tooth; second tergite approximately as long as (*C. cognata* at most 1.2×) posterior width. It can be distinguished from *C. gastrolinae* by the following combination of characters: frons without median longitudinal carina; notaulus absent; areolet receiving vein 2m-cu distinctly basal of its middle; Area superomedia and area petiolaris confluent; ovipositor sheath 1.1–1.2× as long as second tergite; coxae entirely black; tergites 6–8 mainly black. *C. gastrolinae* has the frons with median longitudinal carina; notaulus extending to the middle of the mesoscutum; the areolet receiving vein 2m-cu is placed at its middle; Area superomedia and area petiolaris separated by distinct carina; the ovipositor sheath is 0.8× as long as the second tergite; fore and mid coxae yellow; tergites 6–8 partly yellowish brown.

### The new species can be distinguished from *C. cognata* by the following couplet inserted into Riedel’s (2017) Identification key:

**Table:** 

34	Prepectal carina angled in the area of the sternaulus and divided into a transverse and pleural part, both similar; genal carina obliterated ventrally; ovipositor sheath c. 0.9–1.2× longer than the first tergite; fore tibia leaner, more than 6× longer than wide (as fig. 46)	***C. pleuralis*** ♀♂
–	Prepectal carina continuous without transverse branch or angle in the pleural part; genal carina complete ventrally and arced outwards, meeting the hypostomal carina just before the mandible base; ovipositor sheath c. 0.8–0.95× as long as the first tergite; fore tibia rather swollen, less than 6× longer than wide (fig. 48)	**34**’
34’	Malar space 0.8–0.9× as long as basal width of mandible. Hind tibial spur distinctly shorter than half length of hind first tarsomere. Second tergite 0.9× as long as its apical width. Basal flagellomeres black. Hind tibial spun yellow. All coxae of male black	***C. deserticola* Sheng & Zhou, sp. nov.** ♀♂
–	Malar space 0.6–0.7× as long as basal width of mandible. Hind tibial spur at least 0.5× as long as hind first tarsomere. Second tergite 1.1–1.2× as long as its apical width. Basal flagellomeres yellowish. Hind tibial spun reddish. fore and middle coxae of male yellow	***C. cognata* (Tschek)** ♀♂

## Supplementary Material

XML Treatment for Campoletis

XML Treatment for Campoletis deserticola
